# Duplicate analysis method: a cheaper alternative to commercial IQC materials in limited resource settings for monitoring CD4 testing

**DOI:** 10.1186/s12981-015-0067-6

**Published:** 2015-08-14

**Authors:** Ashwini Shete, Dharmesh P Singh, Bharati Mahajan, Amol Kokare, Ramesh Paranjape, Madhuri Thakar

**Affiliations:** National AIDS Research Institute, 73 G Block MIDC Bhosari, Pune, 411026 India

**Keywords:** CD4, IQC, Duplicate analysis, Commercial controls

## Abstract

**Background:**

India has a large number of HIV infected patients being followed up at anti-retroviral therapy (ART) centers. The patients are regularly offered CD4 count estimation for deciding their eligibility for ART initiation as well as for monitoring response to ART, making CD4 count estimation a very critical test. Hence, quality control of CD4 testing is utmost important for ultimate success of ART program. As the commercial controls are very expensive, internal quality control (IQC), at present, is being done by duplicate analysis method using previous day samples in most of the laboratories. Hence the study was undertaken to review performance of duplicate analysis method for monitoring daily IQC.

**Methods:**

Quality control (QC) data from 11 Indian laboratories using duplicate analysis and/or commercial controls for IQC of CD4 testing was collected for reviewing information on QC parameters such as precision, accuracy and trend monitoring. Precision was determined by r^2^ values and mean % variation for duplicate analysis and coefficient of variation (% CV) for commercial controls. Accuracy was monitored by rate of QC failures for both the types of control and trend monitoring was done by plotting LJ charts for commercial controls and by plotting daily % variation for duplicate analysis.

**Results:**

The laboratories using duplicate analysis for IQC showed good precision with mean % variation ranging from 0.5 to 7.2. There was good match between r^2^ values and % CV of the laboratories performing both the types of QC methods. Rates of QC failures were 2.3 for duplicate analysis and 3 per laboratory-year for IMMUNO-TROL controls. Daily trend monitoring showed fluctuation of daily counts around mean in LJ charts and of percent variation around 0% in duplicate analysis method. Commercially available controls showed limitations such as altered specimen quality leading to difficulties in manual gating and issues with the establishment of laboratory range.

**Conclusion:**

Duplicate analysis can serve as a cheaper alternative to commercially available controls for IQC of CD4 testing especially when supplemented with other QC measures for controlling variations caused by reagent, equipment, staff and environment in addition to the successful participation in External Quality Assurance programme.

## Background

India has been successful in controlling epidemic of the Human Immunodeficiency Virus (HIV) as evident from reduction of adult prevalence from 0.41% in 2001 to 0.27% in 2011 with the current estimated number of people living with HIV/AIDS (PLHIV) being 2.089 million [1]. Additionally, free ART (anti-retroviral therapy) program in India has improved quality of life of people living with HIV/AIDS and has resulted in 29% reduction in estimated annual deaths due to AIDS related causes between 2007 and 2012 [1]. As of March 2014, nearly 1.76 million PLHIV have been registered at 425 ART centres of whom over 0.77 million clinically eligible patients are receiving free ART in Government health facilities [1]. For taking decision on initiation of ART and also for monitoring response to ART in HIV infected patients, currently CD4 lymphocyte enumeration is the only primary laboratory test used in India. Hence reliability of CD4 count report is very important for effectiveness of ART program. CD4 count estimation is performed at around 254 CD4 estimating laboratories and during 2013-14 alone about 15,01,150 CD4 tests were performed [1]. Quality assurance programme for assuring reliability of CD4 counts for all these laboratories is monitored by National AIDS Research Institute (NARI) and National AIDS Control Organization (NACO) through staff trainings, external quality assessment programme, equipment maintenance, kit supplies, etc. Additionally the laboratories also follow internal quality control procedures laid down in their own laboratories.

The main objective of internal quality control (IQC) is to ensure day-to-day consistency of an analytical process [2, 3]. Hence, the focus of IQC is principally on monitoring precision and, to a lesser degree, on accuracy [4]. Under ideal conditions, IQC for CD4 count measurement is done using commercially available stabilized blood samples [5] serving as Certified Reference Materials (CRMs) which are matrix-matched and have assigned target values and ranges for each variable, reliably determined from data obtained by repeated analysis [5, 6]. They usually have open shelf life of 1–3 months [5]. Commercially available stabilized blood samples are recommended for daily QC for CD4 testing as they give information regarding precision, biases and accuracy of the results. Alternative method for QC is duplicate analysis method in which patient samples tested on previous day are used as QC material [7]. This method has two distinct advantages as the QC materials match with the exact nature of the sample (fresh whole blood), and the materials are readily available at any laboratory at no extra cost [6].

The major drawback of commercially available controls is their cost which causes considerable stress on the management system in financial as well as logistic terms especially in settings where the CD4 count is being estimated at such a large scale. Additionally India is moving towards decentralization of HIV treatment services to primary health care (PHC) centers to reduce the burden of providing HIV services on many tertiary care centres and also to enhance access to treatment like in many other countries. It may not be feasible to implement quality control using commercially available controls at PHC level as it would increase the budget by many folds in addition to issues related to their regular supply, transport and storage conditions, compatibility with point of care machines, etc. In India, most of the CD4 laboratories use duplicate analysis method (using previous day’s sample as IQC material), while only some laboratories use the commercial controls for QC monitoring. Hence in the present study, the performance of duplicate analysis method in monitoring QC was reviewed in comparison to that of the commercial control in order to choose a cheaper QC method without compromising the quality. The IQC data from different laboratories in India using either or both the IQC methods was analyzed for parameters of IQC such as precision, accuracy and trend monitoring. The laboratories were selected for getting representative data from different equipments as well as from different geographic locations.

## Results and discussion

### Equipment displays of the processed samples and analysis

**Fig. 1:**
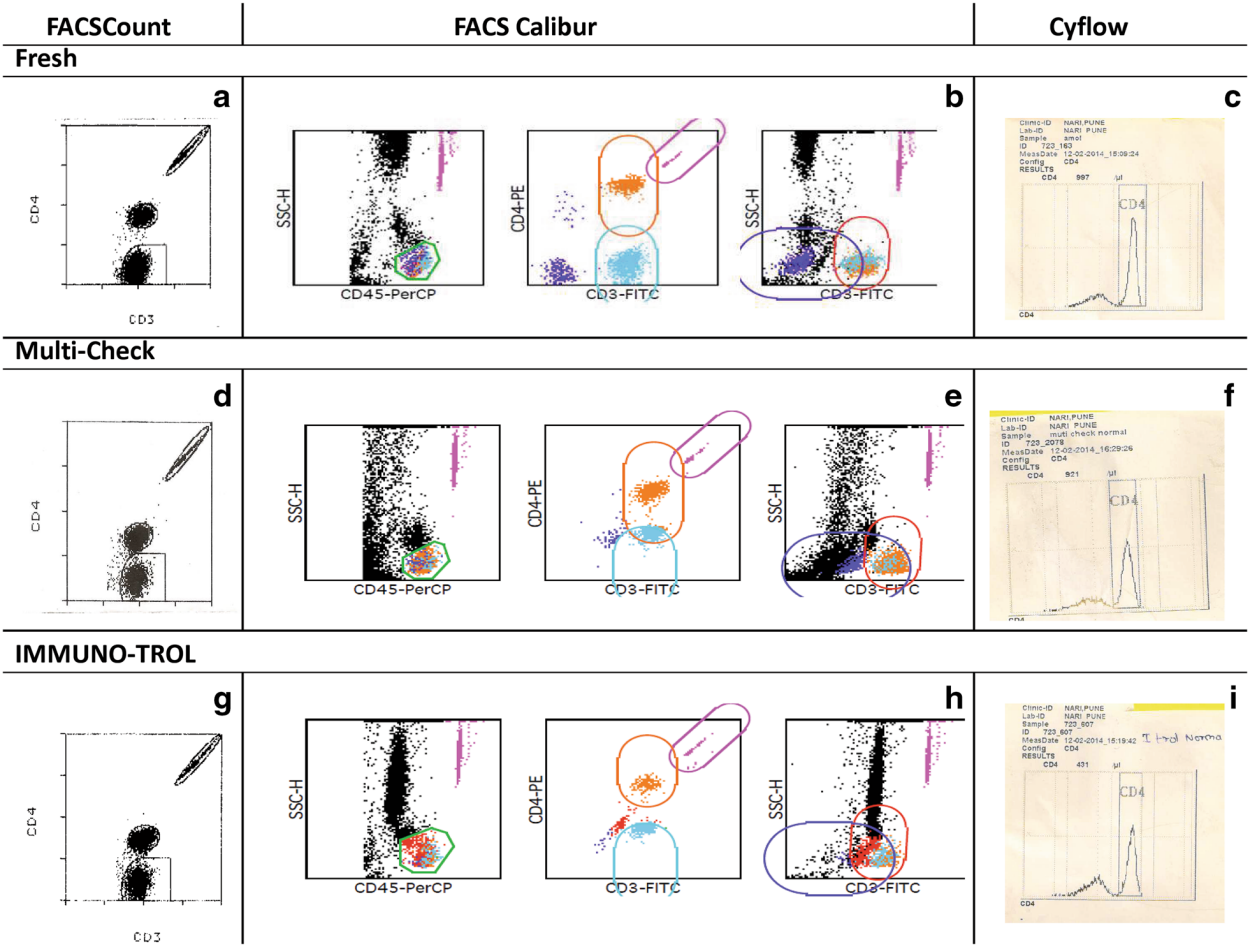
Representative displays of fresh and stabilized blood samples on FACSCount, FACSCalibur and Cyflow. Equipment displays in *row 1* are for fresh blood sample used in duplicate analysis method, *row 2* are for the multi-check stabilized blood sample and *row 3* are for IMMUNO-TROL controls. **a**, **d**, **g** are from FASCount; **b**, **e**, **h** from FACSCalibur and **c**, **f**, **i** from Cyflow. BD FACSCount CD4/CD3 reagent kit contained anti CD3 PE-Cy5 and anti CD4 PE antibodies. Tricolour reagent used for FACSCalibur analysis contained anti CD45 PerCP, anti CD3 FITC and anti CD4 PE antibodies. The *gates* shown in the figure are autogates set by the Multiset software used for FACSCalibur analysis. CD4 easy count kit used for Cyflow contained anti CD4 PE antibody for staining, where the *gates* were set manually.

Displays of commercially stabilized blood samples were found to have poor separation of cell populations as compared to fresh blood samples as shown in Fig. 1. The stabilized blood samples have been shown to have altered light scatter and fluorescence staining properties as compared to fresh blood specimens, not satisfying the automatic gating algorithm defined by the instrument software [8, 9]. Hence manual gating was required in case of FACSCalibur for analysis of stabilized blood samples. Setting of manual gates was found to be challenging because of poor separation of populations, requiring intense training of the staff. Such gating was also found to be subjective and unreliable at many times by other investigators also [9]. FACSCount also sometimes failed to acquire these controls, especially IMMUNO-TROL controls, by giving a message of ‘Major tube failure’. Such testing failure due to inability to identify and gate clusters of cells of interest in case of fully automated platforms like FACSCount has also been reported in one of the studies [9]. Interestingly, the displays on Cyflow did not have much problem of poor separation as shown in Fig. 1.

### Precision monitoring

**Fig. 2:**
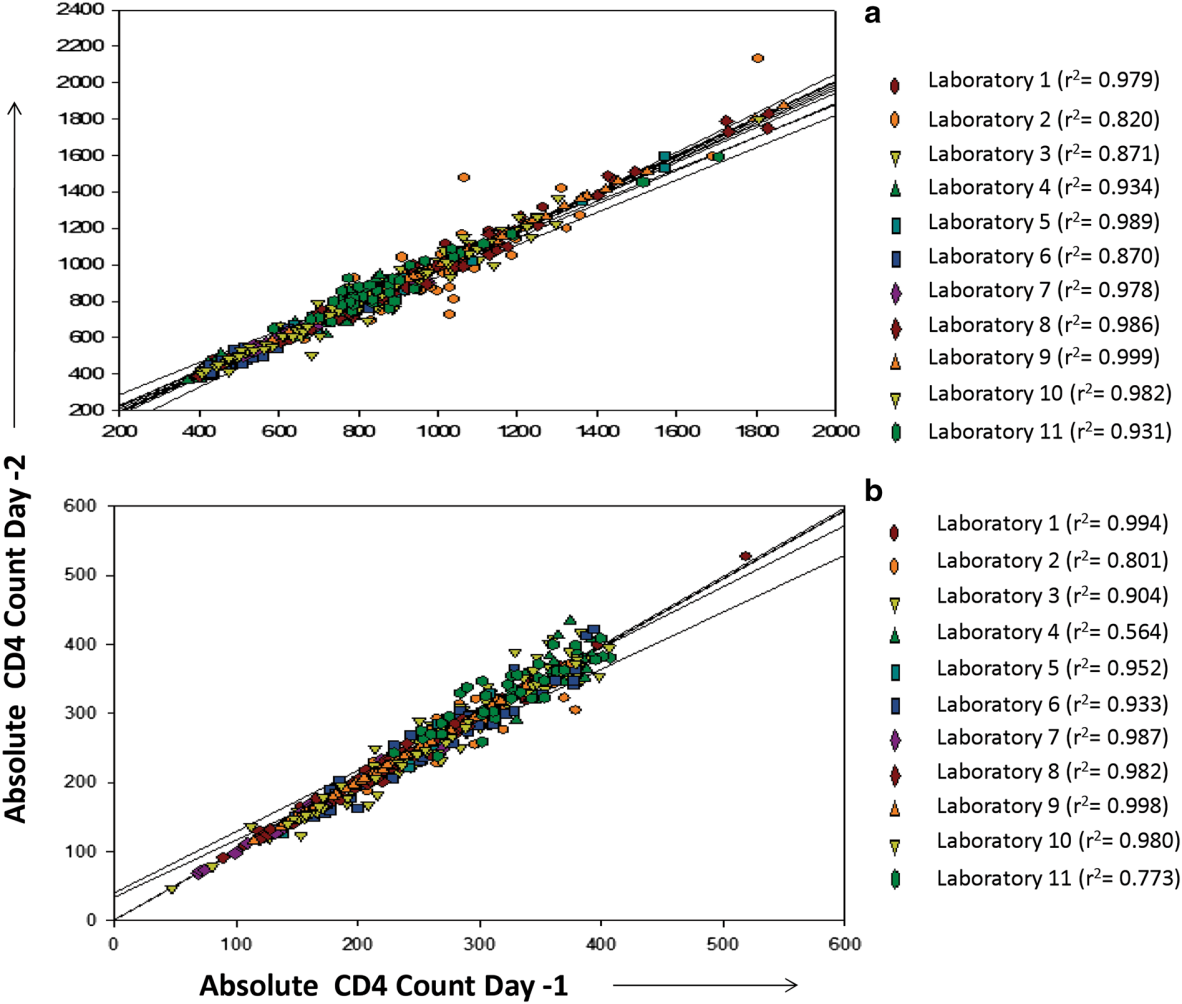
Regression analysis of the CD4^+^ T cell counts in duplicate analysis method. Regression plots for duplicate analysis method plotted for samples with normal CD4^+^ T cell count (**a**), and low CD4^+^ T cell counts (**b**). Day 1 CD4 counts are plotted on X axis and day 2 CD4 counts are plotted on Y axis. Data from different laboratories are presented using different *colours*.

**Fig. 3:**
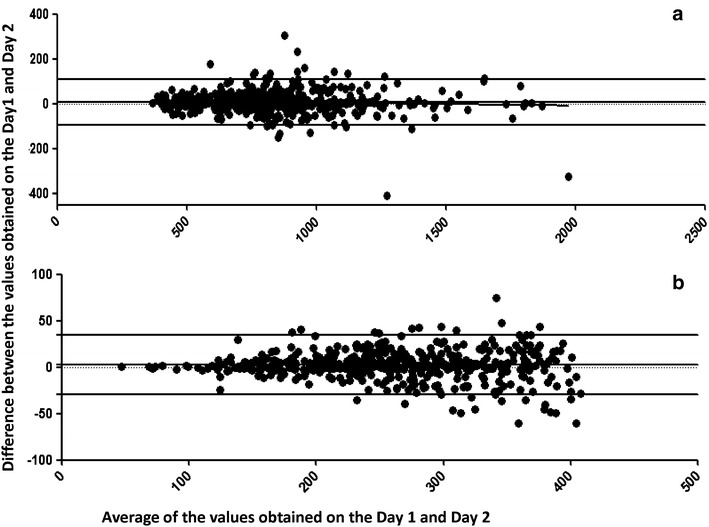
Bland–Altman bias plots for the CD4 values obtained on day 1 and day 2. The figure shows the Bland Altman plots for the duplicate analysis method using samples with normal (**a**) and low (**b**) CD4 counts. The X axis shows the average between the values obtained on both the days and the Y axis shows the differences between the values obtained on the 2 days. The mean differences in CD4 counts, the lower limits of agreement (mean −2 SD), and the upper limits of agreement (mean +2 SD) are displayed as *horizontal lines*.

**Fig. 4:**
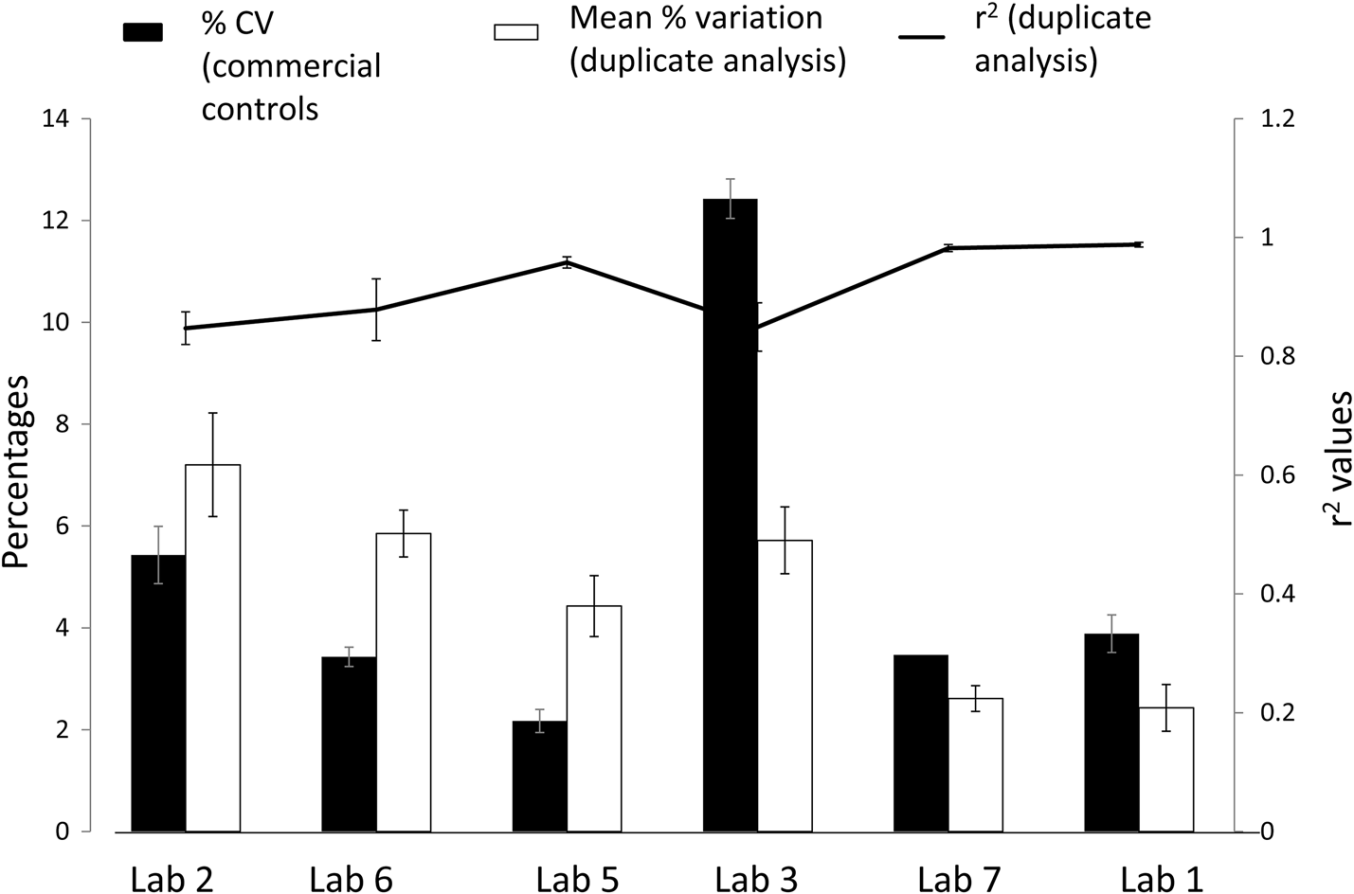
Comparison of % CV, mean percent variation differences and r^2^ values. % CV (calculated for commercial controls) indicated as *closed bars* and mean percent variation (for duplicate analysis method) indicated as *open bars* are plotted on left Y axis for laboratories 1, 2, 3, 5, 6, 7 (plotted on X axis). r^2^ values calculated by regression analysis for duplicate analysis method are indicated as *line diagram* and are plotted on Z axis. *Error bars* for each parameter indicate standard error.

The main objective of IQC is the monitoring of precision [4]. For commercially available controls, precision is determined by calculating % CV. The % CV for the commercial controls ranged from 2.2 to 12.5 (Table 1) for different laboratories. In case of duplicate analysis method, % variation was used for monitoring daily precision. The mean % variation for different laboratories ranged from 0.5 to 7.2 (Table 1). This was well within the acceptable limit mentioned in the NACO and other guidelines. Long term precision in case of the duplicate analyses was determined by calculating r^2^ values and mean of % variation over a period of time. All the laboratories using previous day sample as IQC showed good long term precision as evident from r^2^ value of more than 0.8 when data over 3 months was compared (Fig. 2). Bland–Altman analyses also demonstrated close agreement between day 1 and day 2 CD4 counts, with biases of 8.28 ± 51.4 and 2.38 ± 16.1, for the normal and low level absolute CD4 count controls, respectively (Fig. 3). Where the parallel data on both the controls was available during the same time (N = 6 laboratories), the r^2^ values were found to follow the same trend as the % CVs of commercial controls for the respective laboratories (Fig. 4). The laboratories showing lower % CV values also showed higher r^2^ values and vice a versa showing that the precision of duplicate analysis was comparable to that obtained using commercially available controls. Thus, r^2^ values should also be calculated for duplicate analysis method in addition to % variation on periodic basis to estimate the overall long term precision for the laboratories which will give information similar to that obtained using commercial controls.

**Table 1:** IQC details of CD4 testing laboratories

Laboratories	Instrument used	Mean (range of % variation for duplicate analysis)	Mean (range % CV for commercially available controls)
Laboratory 1	FACSCount	2.43, 0.6–3.3	3.9, 2.7–4.9 (IMMUNO-TROL)
Laboratory 2	FACSCount	7.2, 5.5–12.0	5.4, 3.4–7.0 (multi-check)
Laboratory 3	FACSCalibur	5.7, 2.5–7.2	12.4, 12.0–12.8 (multi-check)
Laboratory 4	FACSCalibur	5.1, 4.2–5.8	–
Laboratory 5	FACSCalibur	4.4, 2.6–6.4	2.2, 1.6–2.6 (multi-check)
Laboratory 6	FACSCalibur	5.9, 4.2–7.0	3.4, 3.0–3.8 (multi-check)
Laboratory 7	FACSCount	2.6, 2.1–3.5	3.5 (multi-check)
Laboratory 8	Cyflow	2.9, 2.0–3.9	–
Laboratory 9	Cyflow	0.5, 0.3–1.2	–
Laboratory 10	Cyflow	3.5, 1.6–5.6	–
Laboratory 11	FACSCalibur	4.8, 3.5–5.3	–

### Accuracy

**Fig. 5:**
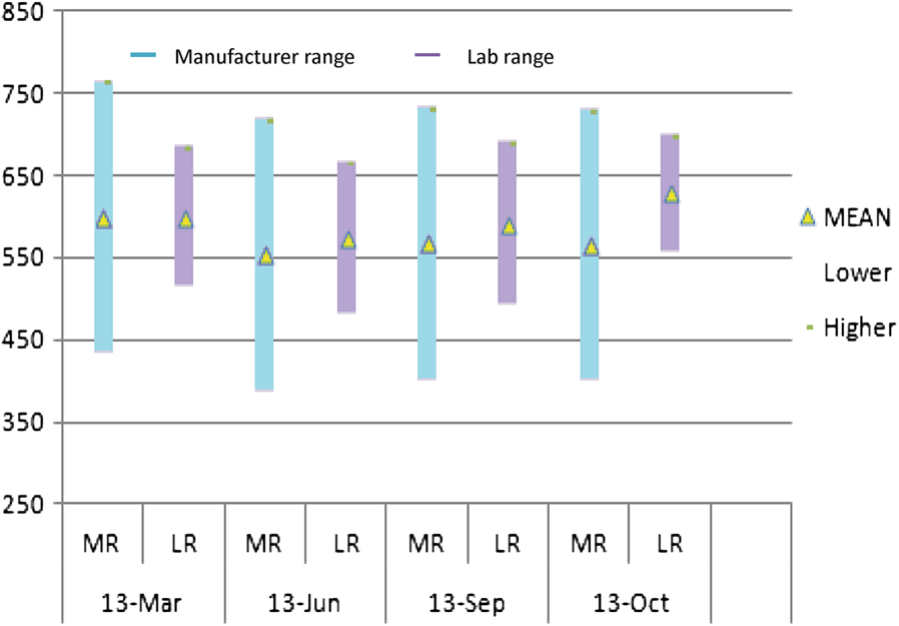
Manufacturer’s range (MR) versus laboratory established ranges (LR) for commercial controls for CD4+ T cells. The ranges (mean ± 2 SD) for different lots of the commercial controls available in different months are plotted. Y axis represents CD4 counts and X axis represents months of the range establishment. *Blue bars* indicate manufacturer’s ranges and *purple bars* indicate laboratory established ranges. The *yellow triangle* in the *middle* of each *bar* indicates mean values.

Since commercially available controls come with manufacturer’s range, they give some idea about the accuracy of the results. But they may not give the exact information about the accuracy as the range is wide as shown in Fig. 5 and it is advisable to establish laboratory range based on its own mean and standard deviation (SD). It was not feasible for most of the laboratories using multi-check controls to establish their own range because of their short expiry leading to false estimate of accuracy. Establishing provisional range based on 10 measurements and then subsequently using 20 data points for establishing the final range [10] would be more appropriate monitoring daily QC in such case.

Duplicate analysis method has also been shown to provide information regarding accuracy if tested continuously [11]. IQC samples showing more than 20% variation would indicate inaccuracy in the testing. The data for the 6 laboratories that used both the types of controls showed average rate of QC failures as 2.3, 0.5, and 3 times laboratory-year, respectively, when duplicate analysis method, multi-check and IMMUNO-TROL controls were used, for which appropriate corrective actions were taken. The duplicate analysis method and the use of IMMUNO-TROL controls showed similar QC failure rates. The lower rate of QC failure with Multi-Check could be because of use of wider manufacturer’s range.

EQA performances are known to provide more exact information regarding accuracy of the testing [4]. All these laboratories had acceptable performance in EQA program conducted nationally indicating it would be sufficient to use duplicate analysis method on continuous basis if the laboratory is successfully participating in regular EQA programme.

### Trend monitoring

**Fig. 6:**
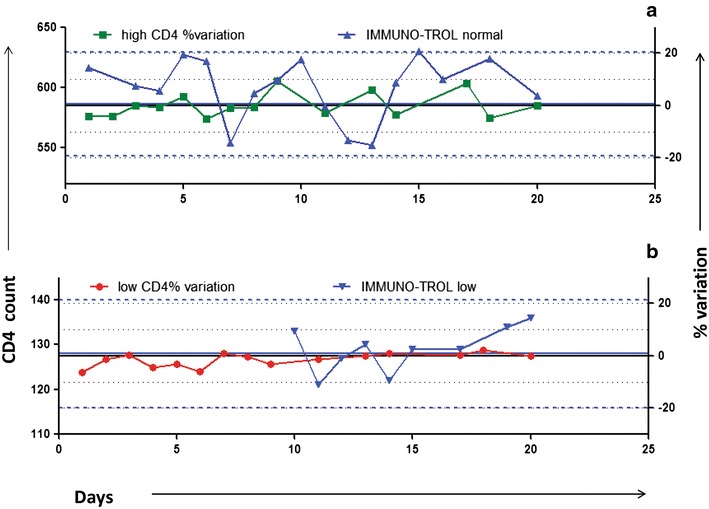
Simultaneous trend monitoring by commercial controls and duplicate analysis. **a** (normal CD4 count), **b** (low CD4 count) show representative *graphs* for simultaneous trend monitoring by duplicate analysis method and by commercial controls by plotting LJ charts. CD4 counts for commercial controls are plotted on left Y axis and % variation as obtained by duplicate analysis method is plotted on right Y axis against the no. of days on X axis. *Green* and *red colour lines* indicate % variation of samples with normal and low CD4 count, respectively. *Black coloured solid line* and *two dotted lines* on either side indicate 20% limits of percent variation, respectively.* Blue line* indicate CD4 counts of IMMUNO-TROL controls with *blue coloured solid line* and *two dotted lines* on either side indicating mean and 2 SD, respectively.

**Fig. 7:**
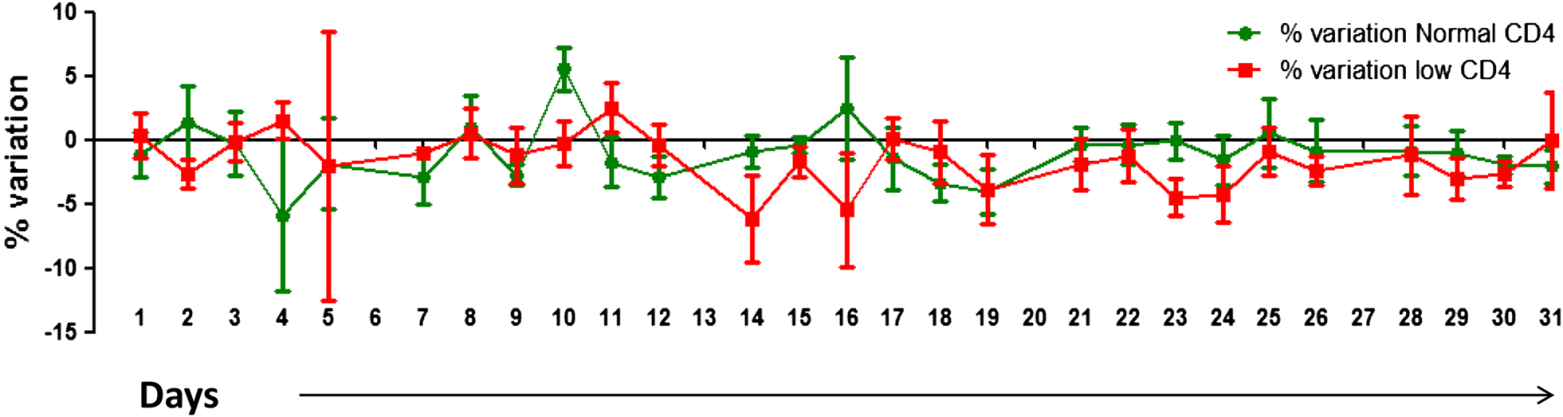
Trend monitoring by duplicate analysis. Fluctuation of daily % variations for monitoring the trend by duplicate analysis method. Mean and SE of % variation of CD4 testing is plotted on Y axis and no. of days are plotted on X axis. % variation for samples with normal CD4 count is shown in *green* colour and that with low CD4 counts is shown in *red* colour.

One more advantage of the commercially available controls is their ability to monitor trend or shifts in the results, which can give the earliest identification of the problems related to changes in reagent lots, technical staff, instrument settings, environmental condition, etc. [12]. However, since most of the laboratories didn’t establish their own ranges because of short expiry of multi-check controls, it was not possible for them to monitor the trend as it is not recommended to use manufacturer’s range for this purpose. Trend monitoring was done by only one laboratory using IMMUNO-TROL control which has a longer shelf life. A representative LJ plot for the laboratory is shown in Fig. 6 along with the simultaneous trend monitoring by duplicate analysis method. Trends and shifts in duplicate analysis method were monitored by deviations of percent variation around 0%. The effective trend monitoring by this method was also stressed previously using continuous method of duplicate analysis [11]. For trend monitoring across CD4 testing laboratories, mean and standard error of daily percent variations was calculated and plotted against the days of the month as shown in Fig. 7. The graph demonstrates that the mean and standard error (SE) of percent variations across the laboratories also fluctuate around 0 %. However, the drawback of duplicate analysis method is that it detects changes only between two successive runs as against the commercial controls which monitor trend over the time. But since all the laboratories implement QC measures for controlling variations because of changes in reagent batches (parallel testing), equipment settings (equipment validation and calibration), staff (training and competency), environment (temperature and environment monitoring), the possible factors leading to trends or shifts in QC data are additionally controlled. Hence the duplicate analysis might be sufficient to monitor the trend if these additional measures are implemented.

### Cost implications

Commercially available controls are largely manufactured outside India and have to be imported and are very expensive. Additionally they have to be run 20 times for establishing their range if they are required to be used optimally. The approximate cost of these reagents per level per month comes to around 100 US dollar, and if 20 runs are performed for range establishment, the cost increases by 2 times as it requires one more tube of the controls as well as CD4 testing reagents, which cost approximately 5–6 USD per test. In the program settings in India it increases by many folds as it includes multiple laboratories all over the country. The cost factor would become more important in case of decentralization of the care as along with the increase in number of centers, the sample load per center would decrease drastically. In duplicate analysis method, all this cost gets nullified making it a cheaper method for broader use.

## Conclusion

Although commercially available controls are recommended for monitoring of daily QC, they have some limitations such as altered sample displays, requirement of laboratory established range for optimal use and factors affecting establishment of laboratory range, etc. Also cost of running these controls is very high, which may not be affordable in resource limited settings. Contrary, duplicate analysis method using previous day samples can monitor QC at no extra cost and may be financially viable alternative, especially in the primary and secondary care settings. The analysis showed that it has a potential to monitor daily as well as long term precision, accuracy as well as trends if done appropriately and continuously. This QC method along with the successful participation in EQAS and institution of other QC measures for controlling variations caused by reagent, equipment, staff and environment would serve as the cost effective system for monitoring quality of CD4 count testing.

## Methods

QC data from 11 laboratories from different regions in India under NACO ART program was collected for analysis during the period of Jan to Dec 2013. CD4 estimating equipments used in these laboratories were FACSCalibur (n = 5), FACSCount (n = 3) (both from Becton–Dickinson, USA) and Cyflow (Partec, Germany) (n = 3) as shown in Table 1. All the laboratories used duplicate analysis method (previous days sample) for daily CD4 QC monitoring. Six of 11 laboratories also used commercially available controls either in addition to or as a substitute to duplicate analysis method for daily QC for variable period ranging from 1 month to entire year. All laboratories performed the procedures as per SOPs and manuals respective to their available equipment. All 11 laboratories successfully participated in the national external quality assurance (EQA) programme for CD4 count estimation for last four years.

### Duplicate analysis

In this method, two whole blood samples collected and processed on previous day having low (200–400 cells/ml) and normal CD4 (>500 cells/ml) count were used for the daily internal quality control. The samples were stored at room temperature for processing on FACScount/FACSCalibur and at 2–8°C for Cyflow as per the manufacturer’s instructions. The CD4 values were compared with the previous day values and percent variation between the values was calculated by using following formula: observed (day 2 value)/expected (day 1 value) × 100 − 100. Percent variation more than ±20 was considered as non acceptable [7].

### Commercially available stabilized controls

Commercially available controls used by the laboratories were IMMUNO-TROL (Beckman coulter, USA) or Multi-Check (BD biosciences, USA). The controls were stored at 2–8°C as per the manufacturer’s instructions and processed along with the samples as per the SOPs to ensure their values were within the prescribed ranges. Most of the laboratories using multi-check controls used manufacturer’s range for monitoring the daily QC as the controls had shorter shelf life of about 1 month and it was not feasible to establish laboratory range for them. For IMMUNO-TROL controls, laboratory established ranges were used after performing 20 runs. LJ charts were plotted daily on the basis of laboratory established ranges for monitoring precision, biases and trends as per the Westgard rules. % CV was calculated at the end of the month for both the commercial controls.

### Data collection and analysis

The QC data as well as representative displays of the processed samples obtained from each of the equipments (FACSCalibur, FACSCount and Cyflow) from the laboratories were collected over the last year for analysis and comparison. Results of QC using commercially available controls and using duplicate analysis method were analysed with respect to equipment displays of the processed samples and for precision, accuracy and trend monitoring. The precision, accuracy and trend was monitored by calculating % CV, out of range readings based on manufacturer or laboratory established ranges and by plotting LJ charts, respectively, in case of commercial controls.

The precision in case of duplicate analysis method was monitored by mean percent variation and r^2^ values for assessing long term precision in addition to the daily percent variations. The mean of daily percent variations was calculated after ignoring minus (−) sign of percent variation over a period of months. The correlations of the CD4 counts on both the days were analyzed by Pearson’s correlation test and regression analysis for calculating r^2^ values. The degree of agreement between the values obtained on 2 days and the biases were estimated using Bland–Altman analysis. Percent variations beyond 20% were unacceptable and considered as QC failure. Rate of QC failures per laboratory-year was calculated by dividing number of QC failures (percent variation >20% for duplicate analysis method or out of range readings for commercial controls) in all laboratories by sum of total months contributed by all laboratories. Mean and standard error (SE) of daily percent variations from CD4 testing laboratories was calculated and plotted against days for trend monitoring by duplicate analysis method. Microsoft Excel, GraphPad prism (version 5) and SigmaPlot (version 12.0) software were used for statistical analysis and plotting graphs.

## Abbreviations

HIV: human immunodeficiency virus
AIDS: acquired immuno deficiency syndrome
ART: anti-retroviral therapy
CD: cluster of differentiation
WHO: World Health Organization
PLHIV: people living with HIV
QC: quality control
IQC: internal quality control
NACO: National AIDS control Organisation
NARI: National AIDS Research Institute
EQA: external quality assessment
EQAS: external quality assessment scheme
FACS: fluorescence activated cell sorting
FITC: fluorescein isothiocyanate
PE: phycoerythrin
PE-Cy5: phycoerythrin-cyanine 5
CV: coefficient of variation
LJ: Levey–Jennings
SD: standard deviation
